# Evaluating the effects of common control measures for influenza A (H1N1) outbreak at school in China: A modeling study

**DOI:** 10.1371/journal.pone.0177672

**Published:** 2017-05-19

**Authors:** Tianmu Chen, Yuanxiu Huang, Ruchun Liu, Zhi Xie, Shuilian Chen, Guoqing Hu

**Affiliations:** 1 Office for Disease Control and Emergency Response, Changsha Center for Disease Control and Prevention, Changsha, China; 2 Department of Epidemiology and Health Statistic, Xiangya School of Public Health, Central South University, Changsha, China; Nanyang Technological University, SINGAPORE

## Abstract

**Background:**

Influenza A (H1N1) outbreaks have become common at schools in China since 2009. However, the effects of common countermeasures for school influenza outbreak have not been quantified so far, including isolation, vaccination, antivirus and school closure. We conducted a mathematically modeling study to address this unsolved issue.

**Methods:**

We collected data of all small-scale school outbreaks caused by influenza A that occurred in Changsha city between January 2009 and December 2013. Two outbreaks (one was in 2009 and the other one was in 2013) were used for simulating the effects of single and combined use of common measures, including isolation (Iso), therapeutics (T), prophylactics (P), vaccinating 70% of susceptible individuals prior to the outbreak (V_P70_), vaccinating 70% of susceptible individuals every day during the outbreak (V_D70_) and school closure of one week (S1w). A susceptible—exposed—infectious/asymptomatic—recovered (SEIR) model was developed to implement the simulations based on the natural history of influenza A.

**Results:**

When no control measures are taken, the influenza is expected to spread quickly at school for the selected outbreak in 2013; the outbreak would last 56 days, and the total attack rate (TAR) would reach up to 46.32% (95% CI: 46.12–46.52). Of all single control measures, V_P70_ is most effective to control the epidemic (TAR = 8.68%), followed by V_P50_, V_D70_, V_D50_ and Iso. The use of V_P70_ with any other measure can reduce TAR to 3.37–14.04% and showed better effects than any other combination of two kinds of measures. The best two-measure combination is ‘S1w+V_P70_’ (TAR = 3.37%, DO = 41 days). All combinations of three kinds of measures were not satisfactory when V_p70_ and V_D70_ were excluded. The most effective three-intervention combination was ‘Iso+S1w+V_P70_’ (with TAR = 3.23%). When V_P70_ or V_D70_ is included, the combinations of four or five kinds of interventions are very effective, reducing TAR to lower than 5%. But the TAR of combination of ‘T+P+Iso+S1w’ is 23.20%. Similar simulation results were observed for the selected outbreak in 2009.

**Conclusion:**

Vaccinating no less than 70% of individuals prior to the outbreak and isolation are recommended as single measures to control H1N1outbreak at school. The combination of V_P70_+S1w can achieve very good control for school outbreak.

## Introduction

Influenza A (H1N1) spreads fast at school and often leads to small-scale outbreaks in China [1]. Ninety percent of influenza outbreaks occurred in schools between 2009 and 2010 in China [2]. In practice, many countermeasures are often taken to control the magnitude of outbreak at schools, including pharmaceutical (typically antivirus and vaccination) and non-pharmaceutical interventions (such as isolation and school closure). However, the impacts of these interventions have not been quantitatively assessed for H1N1 outbreaks at schools so far. In particular, their impacts may differ between small-scale school outbreaks and large-scale epidemic covering a city or across cities in terms of parameter differences between school outbreak and large-scale epidemic such as population density, contact probability, and transmissibility [1]. Without the evidence of common control measures’ effectiveness, it is difficult for public health practitioners to choose appropriate control measures to respond to H1N1 outbreaks at schools. In some cases, empirically arbitrary responses are insufficient or over adequate, leading to unwanted ineffective control or public health resource waste. Thus, it is valuable to conduct a study to quantify the impacts of common control measures on the control of school outbreak in China.

Owing to the lack of epidemic data on non-interventions in real life, it is hard to evaluate the effectiveness of these strategies through traditional epidemiological study designs. Because of this, mathematical modeling has been frequently used in the design and evaluation of influenza control strategies [3–5]. In this study, we collected data of all small-scale outbreaks at schools in Changsha city from January 1, 2009 to December 31, 2013, and used an ordinary differential equation model to evaluate the effectiveness of common countermeasures in school outbreaks, including isolation, vaccination, antivirus and school closure.

## Materials and methods

### Data collection

We built a dataset of influenza A (H1N1) outbreaks by collecting information on all outbreaks at schools reported from 2009 to 2013 in Changsha, China. Information includes type of school (primary school, secondary school, and college or university), size of school population, reporting date of H1N1 outbreak, dates of symptom onset and recovery for all cases, duration of outbreak (DO), and interventions including case isolation, symptomatic treatment of cases, environment disinfection, health education, antivirals for treatment or prophylaxis use, vaccination and school closure (including class, grade and school closure). All data were obtained from the Emergency Public Reporting System and the Influenza Surveillance System. Typically, school influenza outbreaks are reported directly to county CDC by primary health care center, school clinics, or clinics and hospitals when a cluster of influenza-like illness (ILI) cases are observed from the same school. Local CDC identifies school outbreaks directly through daily analysis of reported influenza cases. When an influenza outbreak is confirmed, the school is required to record health status of all students every day and report the information to local CDC until the school outbreak ends [6].

According to the national influenza surveillance guideline [7, 8], ILI refers to having a fever (axillary temperature ≥38°C) accompanied by coughing or sore throat and a lack of a laboratory-confirmed diagnosis of the specific pathogen. In China, an influenza outbreak is defined as ≥ 10 ILI cases occurring in the same school, preschool, or other collective organization within one week [6], along with laboratory-confirmed influenza viruses through virus isolation or real-time reverse transcriptase polymerase chain reaction (RT-PCR) analysis. We selected two moderate school outbreaks (one in 2009 and the other in 2013) from the school outbreak dataset mentioned above to construct mathematical models and estimate major model parameters.

The data was obtained from the Chinese Information System for Diseases Control and Prevention and field epidemiological survey. This data included information on influenza cases and the individuals enrolled in our serosurvey. Written informed consent was given by participants or the adult guardians of children. This study was approved by the Medical Ethics Committee of the Changsha Center for Disease Control and Prevention (CDC). Data can be accessed within the public data management regulation of Changsha CDC. Data are from this study whose authors should be contacted at Mr. Tianmu Chen.

### Model with no intervention

**Fig 1:**
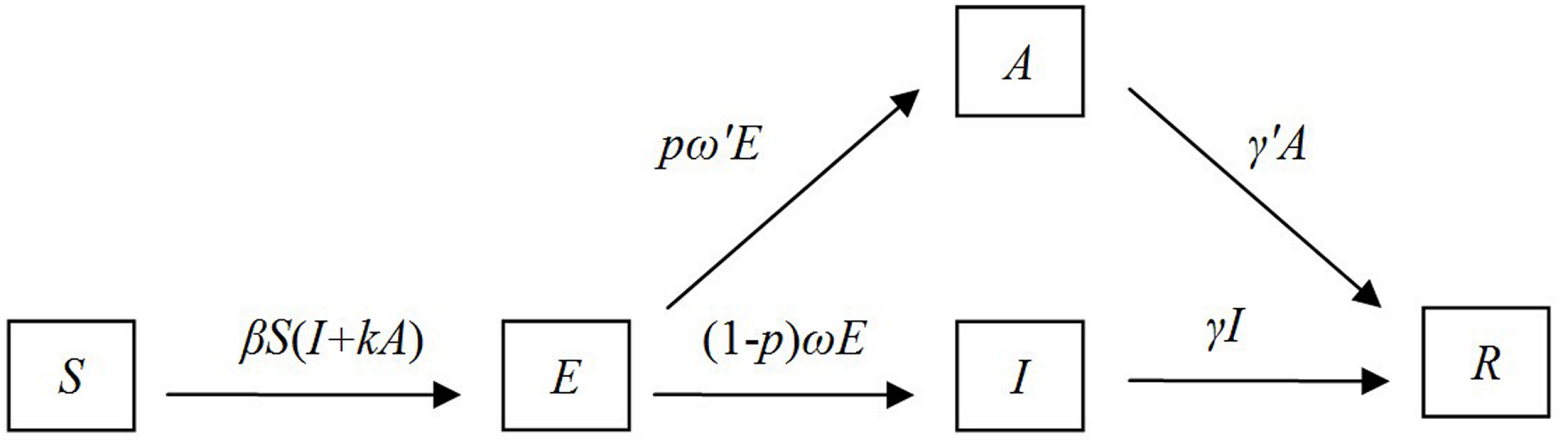
Flowchart of development of the SEIAR model.

According to the natural history of influenza, a susceptible individual is infected by sufficient contact with an ill or asymptomatic person. Newly infected individuals may be asymptomatic or symptomatic but both are infectious in the latent period and infectious state. As the infection progresses, both asymptomatic and symptomatic cases recover with immunity. A typical individual with influenza infection experiences susceptible, exposed, infectious, and recovered phases, with a certain proportion of infected individuals being asymptomatic (Fig 1). A susceptible—exposed—infectious/asymptomatic—recovered (SEIAR) model was reported suitable for simulating an influenza transmission [9]. The SEIAR model can be expressed as the following differential equations:

$$\left\{ \begin{array}{l} dS/dt=-\beta S\left(I+\kappa A\right)\\ dE/dt=\beta S\left(I+\kappa A\right)-p\omega \prime E-(1-p)\omega E\\ dI/dt=\left(1-p\right)\omega E-\gamma I\\ dA/dt=p\omega \prime E-\gamma \prime A\\ dR/dt=\gamma I+\gamma \prime A \end{array}\right.$$ (1)

*S*, *E*, *I*, *A*, and *R* refer to the number of susceptible, exposed, symptomatic, asymptomatic, and removed individuals, respectively. *dS/dt*, *dE/dt*, *dI/dt*, *dA/dt*, and *dR/dt* refer to the changing rates of the *S*, *E*, *I*, *A*, and *R* populations at time *t*, respectively. *β* is the transmission rate from *S* to *E*. *ω* and *γ* refer to changing rates from *E* to *I* and from *I* to *R*, while *ω′* and *γ′* refer to changing rates from *E* to *A* and from *A* to *R*. *κ* and *p* refer to the relative risk of transmission by an asymptomatic individual versus by a symptomatic individual and proportion of asymptomatic infections, respectively (Table 1).

**Table 1:** Parameter definitions and values within the SEIAR model.

Parameter	Description	Unit	Value	Range	Source
*β*	Person–to-person contact rate	1	See text	0–1	Curve fitting
*k*	Relative transmissibility rate of asymptomatic to symptomatic individuals	1	0.5	0–1	References[14–16]
*ω*	Incubation relative rate	day^-1^	0.53	0.14–1	References[14–16]
*ω′*	Latent relative rate	day^-1^	0.83	0.14–1	References[14–16]
*p*	Proportion of the asymptomatic	1	See text	0–1	Serosurvey
*γ*	Recovery rate of the infectious	day^-1^	0.23	0.08–1	References[17]
*γ'*	Recovery rate of the asymptomatic	day^-1^	0.24	0.07–1	References[14–16]

In this model, an equation can be employed to estimate the transmissibility of the virus at schools by the indicator *R* (reproduction number), which was expressed as follows:

$$R=\beta S\left(\frac{1-p}{\gamma }+\frac{\kappa p}{\gamma \prime }\right)$$

### Isolation

In the selected school outbreak, isolation of the symptomatic infected population (*I*) was implemented on November 20, 2013. On the first day of isolation, all symptomatic cases were isolated; after November 20, 2013, any new cases were isolated once they had symptoms. Cases with minor symptoms were requested to stay at home. Dedicated staff paid visits to them to ensure adherence to isolation, environment and hand hygiene, and proper anti-infection. Cases with moderate or severe symptoms were hospitalized and isolated. All influenza cases returned to school when they were free of symptoms for at least two days. In the case-isolation model, the symptomatic-susceptible route is blocked. Nevertheless, individuals in compartment *S* can be infected via the asymptomatic-susceptible contact. We termed the isolated individual *Iso*, and we assumed equal removal rate in isolated individuals as in symptomatic individuals, thus generating the following mathematical model:

$$\left\{ \begin{array}{l} dS/dt=-\beta \kappa AS\\ dE/dt=\beta \kappa AS-p\omega \prime E-(1-p)\omega E\\ dIso/dt=\left(1-p\right)\omega E-\gamma Iso\\ dA/dt=p\omega \prime E-\gamma \prime A\\ dR/dt=\gamma Iso+\gamma \prime A \end{array}\right.$$ (2)

### Vaccination

We simulated two vaccination strategies: vaccination prior to the outbreak (V_P_) and vaccination during the outbreak (V_D_). For the V_P_ strategy, primary public health providers were asked to raise the vaccination rate of school age population before the influenza epidemic season by using the measures like health education and health promotion, thus vaccination effectiveness occurred before the outbreak, and vaccinated individuals were immune to influenza infection during the outbreak. We defined *δ′* as the proportion of V_P_, *N* as the total population, and *S* = (1 − *δ′*) * *N* as the remainder. In China, influenza vaccine coverage remains low [10], especially in student populations. For example, the average coverage rate of trivalent inactivated influenza vaccine was 47.6% among students across 43 schools in 2014–2015 seasons in Beijing [11]. A research by Lv et al showed that the vaccination coverage could be raised greatly through public health policies like health promotion [12]. Therefore, we conservatively assumed that the *δ′* would not exceed 70% in student population in China. In our study, *δ′* was set in four scenarios: 10%, 30%, 50%, and 70%.

**Fig 2:**
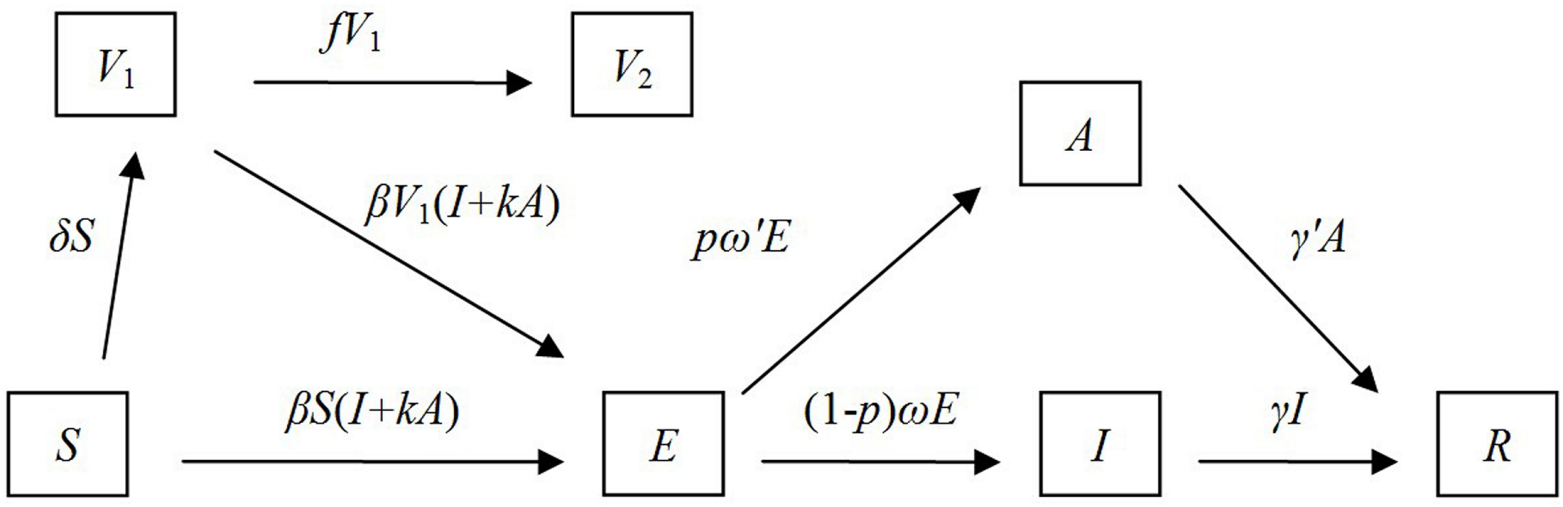
Flowchart of development of the SEIARV model.

The situation was a little complicated for the V_D_ strategy. Supposing that only susceptible individuals were vaccinated, *δ* is considered the daily vaccination proportion, and susceptible individuals receiving vaccination are considered the vaccinated (*V*). Protective antibody will occur within 10 days after a susceptible individual receives a single dose of influenza vaccine [13]. Prior to the formation of antibodies, individuals are subject to influenza infection. *V*_1_ represents vaccinated individuals but still subject to infection, and *V*_2_ represents vaccinated individuals who have developed immunity to infection (Fig 2).

We assumed that individuals in the *V*_2_ population would keep immunity during the outbreak. Before the formation of antibody, population in the *V*_1_ phase has two possibilities of transformations: (1) from *V*_1_ to *V*_2_ at the rate of *f*, and (2) from *V*_1_ to *E* population at the rates of *β* and *κβ* by contacting with *I* or *A*, respectively. Other parameters are assumed to be the same as in the non-intervention SEIAR model. The model with vaccination intervention (SEIARV) was expressed as follows:

$$\left\{ \begin{array}{l} dS/dt=-\beta S(I+\kappa A)-\delta S\\ dE/dt=\beta (S+V_{1})(I+\kappa A)-p\omega \prime E-(1-p)\omega E\\ dI/dt=(1-p)\omega E-\gamma I\\ dA/dt=p\omega \prime E-\gamma \prime A\\ dR/dt=\gamma I+\gamma \prime A\\ dV_{1}/dt=\delta S-\beta V_{1}(I+\kappa A)-fV_{1}\\ dV_{2}/dt=fV_{1} \end{array}\right.$$ (3)

### Antivirals

Currently, oseltamivir is the most commonly recommended antiviral drug for influenza infection in China [6], we evaluated the therapeutic and prophylactic effect of using oseltamivir. A typical five-day course of oseltamivir consists of 10 tablets, 2 per day. The transmissibility of the individual taking oseltamivir needs to multiply by *m* (*m* = 0.38), and the duration from illness onset to recovery need to multiply by *η* (*η* = 0.7658) [14]. The model with therapeutics was expressed as follows:

$$\left\{ \begin{array}{l} dS/dt=-\beta S\left(mI+\kappa A\right)\\ dE/dt=\beta S\left(mI+\kappa A\right)-p\omega \prime E-(1-p)\omega E\\ dI/dt=\left(1-p\right)\omega E-\frac{\gamma }{\eta }I\\ dA/dt=p\omega \prime E-\gamma \prime A\\ dR/dt=\frac{\gamma }{\eta }I+\gamma \prime A \end{array}\right.$$ (4)

For prophylactic use, targeted individuals included *S*, *E*, *A*, and a single course of oseltamivir consisted of 10 tablets (one tablet per day). When taking oseltamivir use into account, the susceptibility of *S* to infection would multiply by *c* (*c* = 0.70), transmissibility of *A* would multiply by *θ* (*θ* = 0.38), and the probability of *E* being infected would multiply by *h* (*h* = 0.4) [14]. The model with prophylactics was expressed as follows:

$$\left\{ \begin{array}{l} dS/dt=-c\beta S\left(I+\kappa \theta A\right)\\ dE/dt=c\beta S\left(I+\kappa \theta A\right)-p\left(1-h\right)\omega \prime E-\left(1-p\right)\left(1-h\right)\omega E-hE\\ dI/dt=\left(1-p\right)\left(1-h\right)\omega E-\gamma I\\ dA/dt=p\left(1-h\right)\omega \prime E-\gamma \prime A\\ dR/dt=\gamma I+\gamma \prime A+hE \end{array}\right.$$ (5)

### School closure

During a school closure, all individuals stay at home. Symptomatic-susceptible and asymptomatic-susceptible contacts are stopped, making *β* to take zero value. We simulated school closures for 1, 2, and 3 weeks, respectively.

### Combinations of multiple interventions

We simulated the following 57 combined interventions to compare their impacts, in which Iso, T, P, V_P70_, V_D70_, and S1w refer to isolation, therapeutics, prophylactics, 70% of individuals vaccinated prior to the outbreak, 70% individuals vaccinated each day during the outbreak, and school closure of one week, respectively. We simulated the effects of all combinations of two, three, four, five, and six kinds of countermeasures, respectively.

### Estimation of parameters

**Fig 3:**
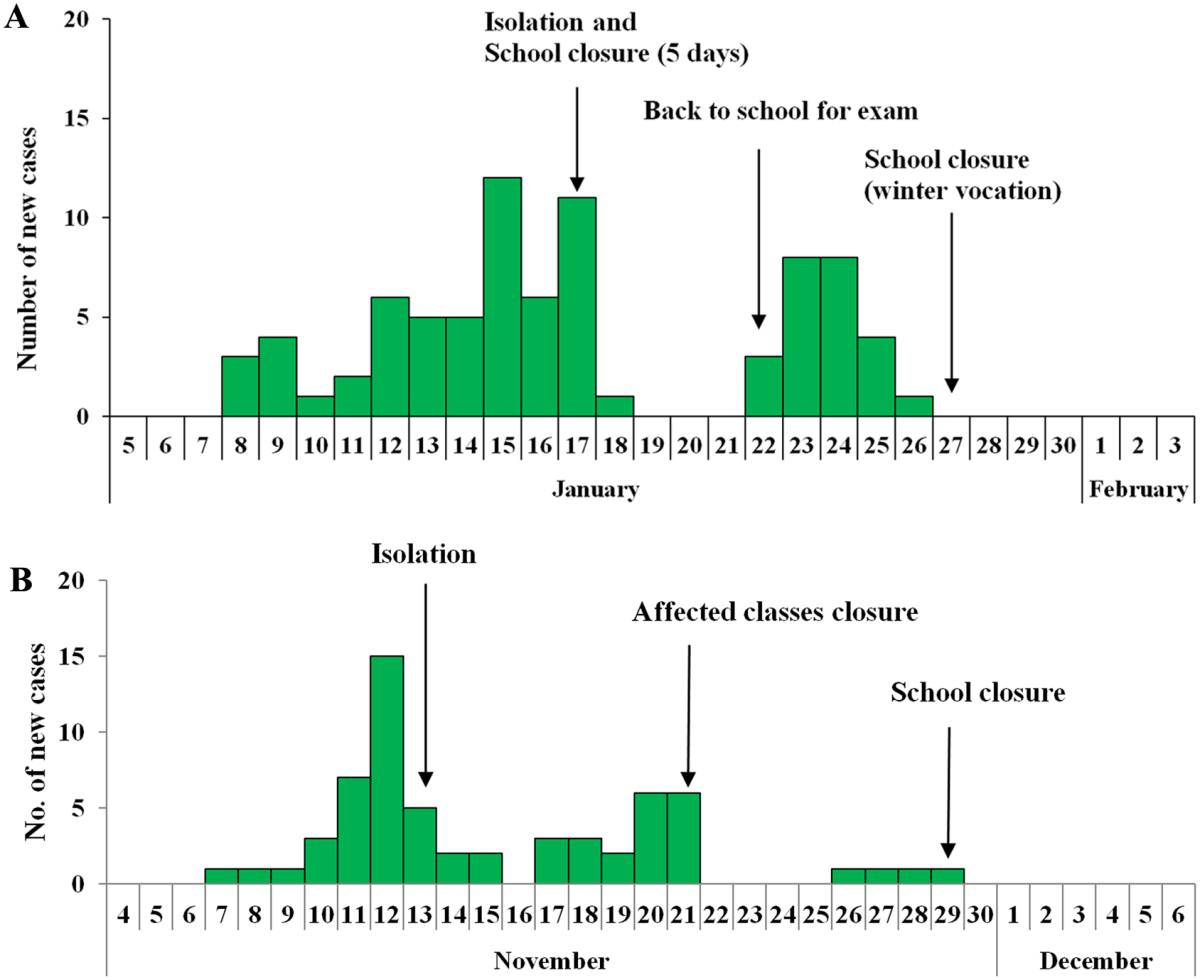
Epidemic curves of two selected influenza A(H1N1) outbreaks at school. A, epidemic curve of influenza A(H1N1) new cases in a middle school, 2013 (The starting date of the outbreak was on January 8, and the duration of the outbreak was 19 days). B, epidemic curve of influenza A(H1N1) new cases in a college, 2009 (The starting date of the outbreak was on November 7, and the duration of the outbreak was 23 days).

Table 1 shows the definitions of parameters and their values in the SEIAR and expanded models. Previously published studies [14–16] suggested the mean influenza incubation periods of 1.9 days (range 1–7 days), mean latent periods of 1.2 days, mean infectious periods of 4.1 days, and half infected peoples as being asymptomatic. Thus we took “*ω* = 0.53, *ω'* = 0.83, *γ'* = 0.24, and *k* = 0.5”. The removal rate of symptomatic individual’s (*γ*), which is the reciprocal of duration from illness onset to recovery, was obtained from the previous publication [17], in which *γ* = 0.23. *β* was estimated using curve fitting with typical events shown in Fig 3.

The parameter *p* was estimated based on the serosurvey in Changsha city, January, 2010. In this survey, 1500 study subjects were selected through multi-stage random sampling. For adults aged 18 years or older, the informed consent was obtained from themselves; for those younger than 18 years, the informed consent form was signed or was obtained by collecting the fingerprint of children and adolescents, or their parents (or adult guardians). The survey questionnaire and the hemagglutination inhibition (HI) assay (a method of antibody titers test) for the serosurvey has been reported described [17]. The serosurvey revealed 1212 of the 1500 respondents not having been vaccinated against influenza A (H1N1), among whom 337 persons were detected having influenza A (H1N1) antibodies. Of the 337 persons, 140 had no influenza-related symptoms between May 2009 and August 2010. We treated the 140 persons as asymptomatically infected individuals, thus having the asymptomatic infection proportion of 41.54%, i.e. *p* = 0.42.

### Simulation methods

Berkeley Madonna 8.3.18 (University of California at Berkeley, Berkeley, USA) and Microsoft Office Excel 2003 software were employed for model simulation and graph plotting, respectively. The Runge-Kutta method of order 4 with the tolerance set at 0.001 was used to perform curve fitting. While the curve fit is in progress, Berkeley Madonna displays the root mean square deviation between the data and current best run [17–19].

### Sensitivity analysis

Considering that four parameters of the SEIAR model, *ω*, *ω'*, *γ'* and *k*, were from the published references and *p* was estimated by a field survey based on only 1500 people, uncertainty may exist for our simulation results. Thus, we did sensitivity analysis by changing five parameters. During the process, the theoretical range of each parameter was split into 1,000 values based on the epidemiological characteristics of seasonal influenza—from 0.14 to 1 for incubation period (1–7 days), from 0.14 to 1 for latent period (1–7 days), from 0.07 to 1 for infectious period of asymptomatic individuals (1–14 days), from 0 to 1 for transmissibility of asymptomatic individuals compared to symptomatic individuals (0–1), and from 0 to 0.77 for *p* it was reported as high as 77% [20], respectively. And the two outbreaks in 2009 and in 2013 were employed to run sensitivity analysis, respectively.

## Results

### Epidemiological features of all outbreaks

Data of 29 influenza A (H1N1) outbreaks at schools was collected in our study (Table 2). 58.62% (17/29) of the outbreaks occurred in secondary school. The proportion of primary school and college or training school both were 20.69% (6/29). The affected population ranged from 126 to 13,485 persons, with the total attack rate (TAR) ranging from 0.72% to 46.03%. There was no significance of the distribution of TAR among primary school, secondary school and college or training school (Kruskal-Wallis test, *χ*^2^ = 2.486, *P* = 0.288). The DO of the 29 outbreaks ranged from 4 days to 43 days, and the differences in distribution of DO among three kinds of schools were insignificant (Kruskal-Wallis test, *χ*^2^ = 1.770, *P* = 0.413). The transmissibility of the 29 outbreaks was high, with the median *R* of 9.24 (range: 2.30–20.22).

**Table 2:** Descriptive characteristics of 29 influenza A(H1N1) outbreaks at schools in Changsha city, China, 2009–2013.

Number	Year	Month	Type of school	Population	DO	Accumulative cases	TAR (%)
1	2009	10	Secondary school	1101	14	44	4.00
2	2009	11	Secondary school	4644	11	143	3.08
3	2009	11	Secondary school	1811	12	77	4.25
4	2009	11	Primary school	1231	11	71	5.77
5	2009	11	Primary school	1028	21	58	5.64
6	2009	11	Secondary school	1874	10	38	2.03
7	2009	11	Secondary school	1192	15	59	4.95
8	2009	11	Secondary school	1342	40	256	19.08
9	2009	11	College	1357	23	61	4.50
10	2009	10	Training school	126	9	58	46.03
11	2009	10	Primary school	1129	14	107	9.48
12	2009	10	College	13485	9	163	1.21
13	2009	11	Secondary school	1240	11	100	8.06
14	2009	11	Secondary school	2050	14	101	4.93
15	2009	11	Secondary school	1138	15	93	8.17
16	2009	11	Primary school	1563	17	42	2.69
17	2009	11	Secondary school	1950	4	18	0.92
18	2009	10	Secondary school	4290	10	31	0.72
19	2009	10	Secondary school	2670	17	95	3.56
20	2009	10	College	2477	7	43	1.74
21	2009	9	College	1434	13	48	3.35
22	2009	11	Primary school	1081	21	155	14.34
23	2009	10	Secondary school	588	15	37	6.29
24	2009	11	Primary school	429	10	19	4.43
25	2009	11	Secondary school	2280	43	96	4.21
26	2009	11	Secondary school	1251	20	127	10.15
27	2009	9	Secondary school	4032	15	60	1.49
28	2009	11	College	2255	15	49	2.17
29	2013	1	Secondary school	2500	19	80	3.20

DO: duration of outbreak; TAR: total attack rate.

From Table 3, we found that primary public health providers in China generally preferred to using non-pharmaceutical (case isolation, health education, environment disinfection and school closure) countermeasures compared to pharmaceutical ones (antivirals and vaccination). Symptomatic treatment by medication (not antivirals) to cases with ILI symptoms was also frequently used in practice. Case isolation and symptomatic treatment were used in each outbreak. Environment disinfection and health education were adopted in 28 outbreaks. School closure was employed in 21 outbreaks. Vaccination during the outbreak was employed in 3 outbreaks. Antivirals and vaccination prior to outbreak were absent in all outbreaks.

**Table 3:** The reproduction number and interventions in each outbreak at schools in Changsha city, China, 2009–2013.

Outbreak ID	*Β*	*R*	Isolation	ST	ED	Health education	Prophylaxis	V_P_	V_D_	School closure
1	5.48×10^−3^	20.22	Yes	Yes	Yes	Yes	No	No	No	No
2	7.28×10^−4^	11.32	Yes	Yes	Yes	Yes	No	No	No	Yes
3	1.28×10^−3^	7.82	Yes	Yes	Yes	Yes	No	No	No	Yes
4	4.34×10^−3^	17.88	Yes	Yes	Yes	Yes	No	No	No	No
5	2.05×10^−3^	7.07	Yes	Yes	Yes	Yes	No	No	No	Yes
6	2.81×10^−3^	17.62	Yes	Yes	Yes	Yes	No	No	Yes	Yes
7	1.91×10^−3^	7.62	Yes	Yes	No	No	No	No	No	Yes
8	1.48×10^−3^	6.66	Yes	Yes	Yes	Yes	No	No	No	Yes
9	2.04×10^−3^	9.26	Yes	Yes	Yes	Yes	No	No	No	Yes
10	4.33×10^−2^	18.26	Yes	Yes	Yes	Yes	No	No	No	Yes
11	2.42×10^−3^	9.14	Yes	Yes	Yes	Yes	No	No	No	Yes
12	1.15×10^−4^	5.17	Yes	Yes	Yes	Yes	No	No	No	No
13	1.27×10^−3^	5.27	Yes	Yes	Yes	Yes	No	No	No	No
14	2.23×10^−3^	15.30	Yes	Yes	Yes	Yes	No	No	No	No
15	2.32×10^−3^	8.83	Yes	Yes	Yes	Yes	No	No	No	Yes
16	9.19×10^−4^	4.81	Yes	Yes	Yes	Yes	No	No	Yes	Yes
17	3.06×10^−3^	19.95	Yes	Yes	Yes	Yes	No	No	No	Yes
18	6.43×10^−4^	9.24	Yes	Yes	Yes	Yes	No	No	No	No
19	1.18×10^−3^	10.51	Yes	Yes	Yes	Yes	No	No	Yes	Yes
20	9.83×10^−4^	8.15	Yes	Yes	Yes	Yes	No	No	No	Yes
21	1.19×10^−3^	5.70	Yes	Yes	Yes	Yes	No	No	No	Yes
22	3.70×10^−3^	13.38	Yes	Yes	Yes	Yes	No	No	No	Yes
23	9.91×10^−3^	19.50	Yes	Yes	Yes	Yes	No	No	No	Yes
24	7.33×10^−3^	10.53	Yes	Yes	Yes	Yes	No	No	No	No
25	3.01×10^−4^	2.30	Yes	Yes	Yes	Yes	No	No	No	Yes
26	4.40×10^−3^	18.41	Yes	Yes	Yes	Yes	No	No	No	Yes
27	9.13×10^−4^	12.33	Yes	Yes	Yes	Yes	No	No	No	Yes
28	7.18×10^−4^	5.42	Yes	Yes	Yes	Yes	No	No	No	No
29	5.26×10^−3^	4.41	Yes	Yes	Yes	Yes	No	No	No	Yes

ID: Identification; *R*: reproduction number; ST: symptomatic treatment; ED: environment disinfection; V_P_: vaccination prior to the outbreak; V_D_: vaccination during the outbreak.

### The outbreak in 2009

#### Data analysis and curve fitting

Of school outbreaks of 2009, an outbreak in a college was randomly selected to assess the impacts of included interventions on the control of school outbreak. On November 13, 2009, a local branch of the CDC reported an influenza outbreak in a college of 1357 students. The index cases developed symptoms on November 7, after then new cases occurred gradually. On November 13, local CDC implemented a standard investigation. Throat swabs for 16 cases were collected from November 14 to 19, of which 14 were H1N1 positive according to the PCR test by Changsha CDC. On November 13, interventions, including case isolation and supplementary measures (environmental disinfection, ventilation, health education, and hand hygiene) were implemented together. The number of cases started to descend at the same day. New cases increased again and reached a second epidemic peak on November 20, which indicated merely the measures that were adopted in early stage did not well curb the development of outbreak. On November 21, the college closed the classes having more than 2 cases with ILI symptoms. On November 29, the whole college was closed; the last case was reported by the college at the same day. This outbreak had 61 cases in total and a TAR of 4.50% (Fig 3 and S1 File).

**Fig 4:**
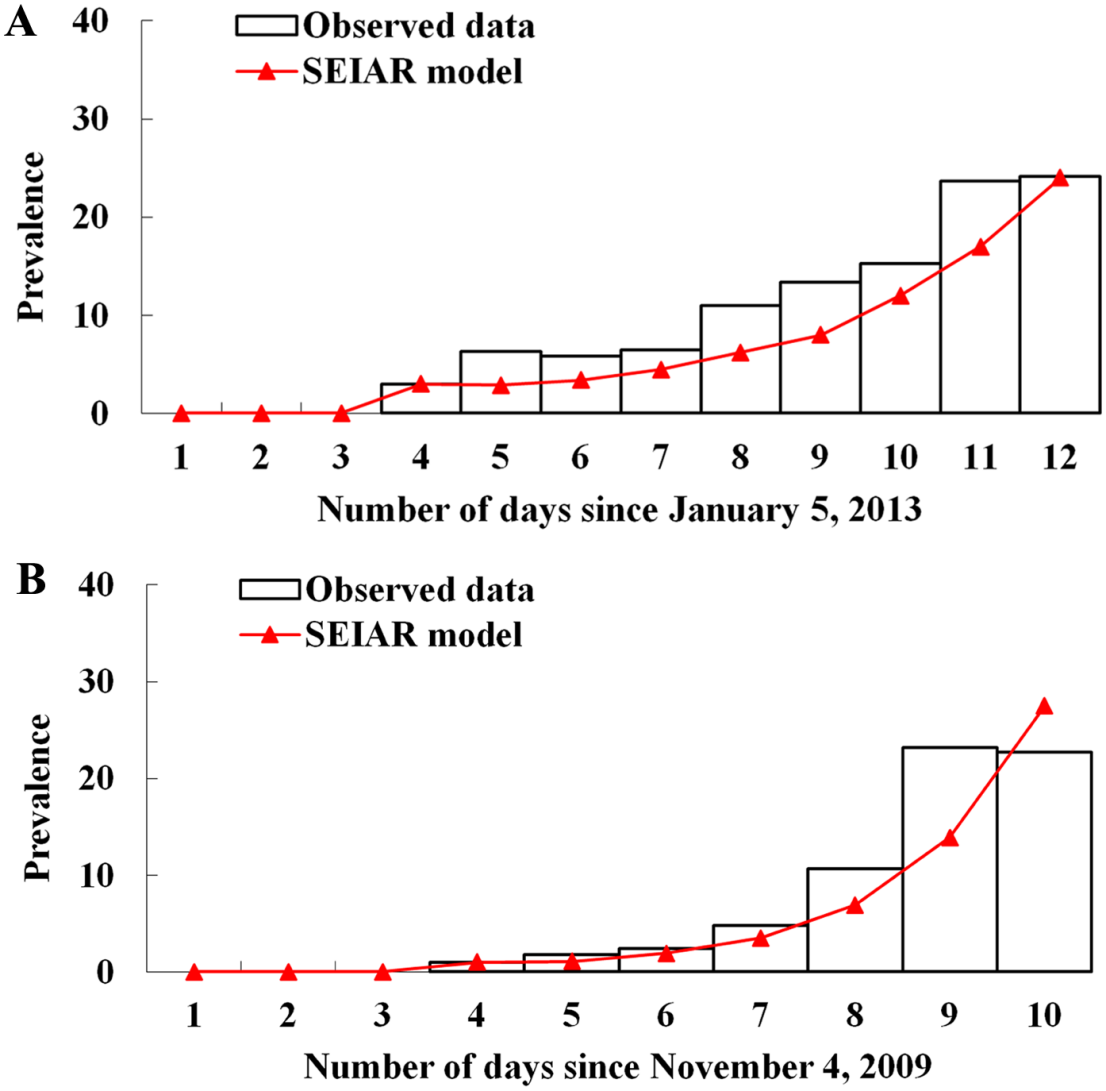
Curve fitting of two selected outbreaks with SEIAR model. A, curve fitting of data from the baseline of the outbreak simulation from January 8 to 16, 2013. B, curve fitting of data from the baseline of the outbreak simulation from November 7 to 13, 2009. Prevalence = I/N, where I is the infectious and N is the total number of persons.

In terms of the implementation time period, we divided the whole epidemic process into two phases: November 7 to 13 (day 4 to day 10), November 14 to the end of the outbreak. SEIAR model was employed to run the curve fitting. The mathematical model showed the best fit (*χ*^2^ = 10.213, *P* = 0.116) to daily reported influenza cases data when *β* taking the value of 2.04 × 10^−3^ (Fig 4).

#### Effectiveness of interventions

With no intervention, influenza spread quickly in the school. The outbreak would last 41 days, and the TAR would reach up to 58.46% (95% CI: 58.05–58.87). The most effective single-intervention strategy was V_P70_, with TAR as low as 15.81% and duration of outbreak (DO) of 61 days. However, the effectiveness of one-, two- and three-week school closure was unsatisfactory. The TARs of these three strategies were close to that for no intervention, and the DO was prolonged (Table 4).

**Table 4:** Simulated effectiveness of single intervention in two selected outbreaks.

Intervention	The outbreak in 2013		The outbreak in 2009	
	TAR % (95%CI)	DO (days)	TAR % (95%CI)	DO (days)
None	46.32 (46.12–46.52)	56	58.46 (58.05–58.87)	41
Therapeutics	40.08 (39.89–40.27)	73	57.90 (57.50–58.30)	39
Prophylactics	42.14 (41.95–42.33)	67	52.30 (51.91–52.68)	52
Isolation	27.80 (27.62–27.98)	105	55.27 (54.88–55.67)	53
School closure				
1 week	46.02 (45.82–46.22)	67	58.46 (58.05–58.87)	50
2 weeks	45.97 (45.77–46.17)	79	58.42 (58.01–58.82)	59
3 weeks	45.97 (45.77–46.17)	90	58.42 (58.01–58.82)	69
Vaccination (Prior to the outbreak)				
10%	41.17 (40.98–41.36)	57	52.57 (52.19–52.96)	41
30%	30.72 (30.54–30.90)	61	40.84 (40.50–41.18)	43
50%	19.55 (19.39–19.71)	69	28.80 (28.51–29.08)	47
70%	8.68 (8.57–8.79)	65	15.81 (15.60–16.03)	61
Vaccination (During the outbreak)				
10%	37.68 (37.49–37.87)	53	55.53 (55.13–55.93)	40
30%	30.43 (30.25–30.61)	51	49.89 (49.51–50.26)	37
50%	27.56 (27.38–27.74)	50	45.11 (44.75–45.46)	34
70%	26.10 (25.93–26.27)	50	41.44 (41.10–41.79)	33

DO: duration of outbreak; TAR: total attack rate.

The two-combined intervention strategies, including all combinations with V_P70_, were effective. The most effective strategy was P+V_P70_, whose TAR was 11.08%, and DO was 35 days. Interestingly, Iso+P also had low TAR (11.07%), but its DO was as long as 37 days. The least effective combination was S1w+T, with a TAR of 57.64% and a DO of 50 days (Table 5).

**Table 5:** Simulated effectiveness of 2- and 3-intervention combinations in two selected outbreaks.

Intervention combinations	The outbreak in 2013		The outbreak in 2009	
	TAR % (95%CI)	DO (days)	TAR % (95%CI)	DO (days)
S1w+V_P70_	3.37 (3.30–3.44)	41	15.77 (15.56–15.98)	75
Iso+V_P70_	4.51 (4.43–4.59)	42	11.25 (11.07–11.43)	42
P+V_P70_	5.35 (5.26–5.44)	82	11.08 (10.90–11.25)	35
T+V_P70_	5.46 (5.37–5.55)	45	13.66 (13.46–13.86)	40
V_P70_+V_D70_	5.78 (5.69–5.87)	40	12.37 (12.18–12.56)	29
S1w+V_D70_	6.12 (6.03–6.21)	50	11.29 (11.11–11.47)	28
Iso+V_D70_	8.32 (8.21–8.43)	45	20.72 (20.48–20.96)	30
P+V_D70_	11.51 (11.38–11.64)	51	25.42 (25.15–25.69)	28
T+V_D70_	14.04 (13.90–14.18)	44	29.55 (29.26–29.84)	26
Iso+P	22.86 (22.70–23.02)	169	11.07 (10.89–11.25)	37
S1w+Iso	24.33 (24.16–24.50)	136	54.28 (53.89–54.67)	67
Iso+T	27.80 (27.62–27.98)	104	55.32 (54.92–55.71)	48
T+P	37.15 (36.96–37.34)	93	25.20 (24.93–25.47)	54
S1w+T	39.01 (38.82–39.20)	72	57.64 (57.24–58.05)	50
S1w+P	45.52 (45.32–45.72)	71	49.80 (49.43–50.18)	71
Iso+S1w+V_P70_	3.23 (3.16–3.30)	40	8.36 (8.21–8.52)	45
P+Iso+V_P70_	3.31 (3.24–3.38)	36	7.46 (7.31–7.60)	27
T+P+V_P70_	3.60 (3.53–3.67)	38	8.45 (8.29–8.60)	25
S1w+V_P70_+V_D70_	3.66 (3.59–3.73)	37	7.57 (7.42–7.71)	28
P+Iso+V_D70_	3.79 (3.72–3.86)	38	9.66 (9.49–9.82)	26
Iso+S1w+V_D70_	3.83 (3.75–3.91)	39	8.25 (8.09–8.40)	28
T+S1w+V_P70_	3.85 (3.77–3.93)	42	9.96 (9.79–10.13)	55
P+V_P70_+V_D70_	3.97 (3.89–4.05)	37	9.79 (9.62–9.95)	26
Iso+V_P70_+V_D70_	4.08 (4.00–4.16)	35	8.93 (8.77–9.09)	28
T+S1w+V_D70_	4.20 (4.12–4.28)	39	8.89 (8.73–9.05)	23
P+S1w+V_D70_	4.48 (4.40–4.56)	49	8.97 (8.81–9.13)	26
T+Iso+V_P70_	4.50 (4.42–4.58)	38	11.07 (10.89–11.25)	39
T+V_P70_+V_D70_	4.57 (4.49–4.65)	33	10.09 (9.92–10.26)	24
P+S1w+V_P70_	4.68 (4.60–4.76)	98	7.99 (7.84–8.15)	36
T+P+V_D70_	5.30 (5.21–5.39)	40	10.21 (10.04–10.38)	22
T+Iso+V_D70_	8.32 (8.21–8.43)	41	20.48 (20.24–20.72)	25
T+P+Iso	22.84 (22.68–23.00)	163	10.89 (10.72–11.07)	33
P+Iso+S1w	23.22 (23.05–23.39)	175	7.65 (7.50–7.80)	33
T+Iso+S1w	24.30 (24.13–24.47)	146	53.89 (53.50–54.28)	63
T+P+S1w	38.44 (38.25–38.63)	97	12.60 (12.42–12.79)	64

DO: duration of outbreak; TAR: total attack rate; Iso: isolation; T: therapeutics; P: prophylactics; V_P70_: 70% of individuals vaccinated prior to the outbreak; V_D70_: 70% individuals vaccinated each day during the outbreak; S1w: school closure for one week.

Most of the three- intervention combinations were very effective. The most effective was P+Iso+V_P70_, for which the TAR was 7.46% and the DO was 27 days. Other effective three-intervention strategies consisted of S1w+V_P70_+V_D70_, P+Iso+S1w and P+S1w+V_P70_. The least effective was T+Iso+S1w, for which the TAR was as high as 53.89% and had a DO of 63 days (Table 5).

All other four-, five-, and six-intervention combinations achieved very good control of influenza outbreak, with a DO ranging from 21 to 41 days and a TAR changing from 6.90% to 9.82%. The effectiveness of all strategies involving vaccination was close to each other (Table 6).

**Table 6:** Simulated effectiveness of 4-, 5- and 6-intervention combinations in two selected outbreaks.

Intervention	The outbreak in 2013		The outbreak in 2009	
	TAR % (95%CI)	DO (days)	TAR % (95%CI)	DO (days)
P+Iso+S1w+V_P70_	3.20 (3.13–3.27)	36	6.94 (6.80–7.08)	27
P+Iso+V_P70_+V_D70_	3.23 (3.16–3.30)	31	7.41 (7.27–7.56)	26
P+S1w+V_P70_+ V_D70_	3.25 (3.18–3.32)	34	7.28 (7.14–7.43)	26
P+Iso+S1w+ V_D70_	3.28 (3.21–3.35)	36	7.19 (7.05–7.34)	26
T+P+S1w+V_P70_	3.29 (3.22–3.36)	36	7.11 (6.97–7.25)	24
T+P+Iso+V_P70_	3.30 (3.23–3.37)	32	7.46 (7.31–7.60)	23
T+P+S1w+ V_D70_	3.42 (3.35–3.49)	36	7.63 (7.48–7.77)	21
T+P+V_P70_+ V_D70_	3.43 (3.36–3.50)	29	8.28 (8.12–8.43)	21
Iso+S1w+V_P70_+ V_D70_	3.45 (3.38–3.52)	33	7.16 (7.01–7.30)	28
T+S1w+V_P70_+ V_D70_	3.49 (3.42–3.56)	30	7.28 (7.14–7.43)	23
T+Iso+S1w+V_P70_	3.63 (3.56–3.70)	36	8.36 (8.21–8.52)	41
T+P+Iso+ V_D70_	3.78 (3.71–3.85)	34	9.82 (9.66–9.99)	22
T+Iso+S1w+ V_D70_	3.83 (3.75–3.91)	36	8.40 (8.25–8.55)	23
T+Iso+V_P70_+ V_D70_	4.08 (4.00–4.16)	31	9.09 (8.93–9.25)	23
T+P+Iso+S1w	23.20 (23.03–23.37)	169	7.80 (7.65–7.95)	29
P+Iso+S1w+V_P70_+V_D70_	3.13 (3.06–3.20)	31	6.90 (6.76–7.04)	26
P+T+ S1w+V_P70_+ V_D70_	3.15 (3.08–3.22)	27	6.98 (6.84–7.12)	21
T+P+Iso+S1w+V_P70_	3.19 (3.12–3.26)	31	6.98 (6.84–7.12)	22
T+P+Iso+ V_P70_+ V_D70_	3.22 (3.15–3.29)	27	7.41 (7.27–7.56)	21
T+P+Iso+S1w+ V_D70_	3.28 (3.21–3.35)	32	7.19 (7.05–7.34)	21
T+Iso+S1w+V_P70_+ V_D70_	3.45 (3.38–3.52)	29	7.16 (7.01–7.30)	23
T+P+Iso+S1w+V_P70_+V_D70_	3.13 (3.06–3.20)	27	6.90 (6.76–7.04)	21

DO: duration of outbreak; TAR: total attack rate; Iso: isolation; T: therapeutics; P: prophylactics; V_P70_: 70% of individuals vaccinated prior to the outbreak; V_D70_: 70% individuals vaccinated each day during the outbreak; S1w: school closure for one week.

### The outbreak in 2013

#### Data analysis and curve fitting

A moderate outbreak in a middle school was selected to assess the impacts of included interventions on the control of school outbreak. Data of this outbreak was used to estimate main parameters of mathematical models. On January 16, 2013, a local branch of the CDC reported an influenza outbreak in a middle school of about 2,500 students. The first three cases developed symptoms on January 8, after then new cases occurred gradually. On January 17, local CDC implemented a standard investigation according to the requirement of “Guidelines for Dispose of Influenza-like Illness Outbreak of China (2012 edition)” [6] and “Influenza surveillance program of China (2010 edition)” [7] that are issued by the National Health and Family Planning Commission of the People’s Republic of China. Throat swabs for 11 cases were collected on January 17, of which two were H1N1 positive according to the PCR test by Changsha CDC. On January 17, interventions, including case isolation, school closure for 5 days, supplementary measures (environmental disinfection, ventilation, health education, and hand hygiene) were implemented together. The number of cases started to descend from January 18, 2013. New cases increased again when the students returned to school on January 22, 2013 for a 3-day school exam. As the winter vocation came on January 26, 2013, school-based reporting stopped, with the last case being reported by the school on the same day. This outbreak with 80 cases had a total attack rate (TAR) of 3.20% (Fig 3 and S2 File). The definition of a case was from the Diagnosis and Treatment Guidelines for Influenza (2011 edition) [8].

In terms of the implementation time period, we divided the whole epidemic process into two phases: January 8 to 16 (day 4 to day 12), January 16 to the end of the outbreak. SEIAR model was employed to run the curve fitting. The mathematical model showed the best fit (*χ*^2^ = 32.393, *P* = 0.447) to daily reported influenza cases data when *β* taking the value of 5.26 × 10^−4^ (Fig 4). The model thus reproduced the typical epidemic curve observed for an influenza A (H1N1) outbreak in a school population.

#### Effectiveness of interventions

With no intervention, influenza spread quickly in the school. The outbreak would last 56 days, and the TAR would reach up to 46.32% (95% CI: 46.12–46.52). The most effective single-intervention strategy was V_P70_, with TAR as low as 8.68% and duration of outbreak (DO) of 65 days. However, the effectiveness of one-, two- and three-week school closure was unsatisfactory. The TARs of these three strategies were close to that for no intervention, and the DO was prolonged (Table 4).

The two-combined intervention strategies, including all combinations with V_P70_, were effective. The most effective strategy was S1w+V_P70_, whose TAR was 3.37%, and DO was 41 days. P+V_P70_ decreased TAR to 5.35%, but its DO was as long as 82 days. Other effective two-intervention combinations included Iso+V_P70_, T+V_P70_, V_P70_+V_D70_ and S1w+V_D70_. The least effective combination was S1w+P, with a TAR of 45.52% and a DO of 71 days (Table 5).

Most of the three- intervention combinations were very effective. The most effective was Iso+S1w+V_P70_, for which the TAR was 3.23% and the DO was 40 days. Other effective three-intervention strategies consisted of T+P+V_P70_, P+Iso+V_P70_, S1w+V_P70_+V_D70_, and P+Iso+V_D70_, whose TARs were similar to that for P+S1w+V_P70_, but the DOs were much longer than that of the best combination. The least effective was T+P+S1w, for which the TAR was as high as 38.88% and had a DO of 97 days that was more than twice that for no intervention (Table 5).

Except T+P+Iso+S1w, all other four-, five-, and six-intervention combinations achieved very good control of influenza outbreak, with a DO ranging from 27 to 36 days and a TAR changing from 3.13% to 4.08%. The effectiveness of all strategies involving vaccination was close to each other (Table 6).

### Sensitivity analysis

**Fig 5:**
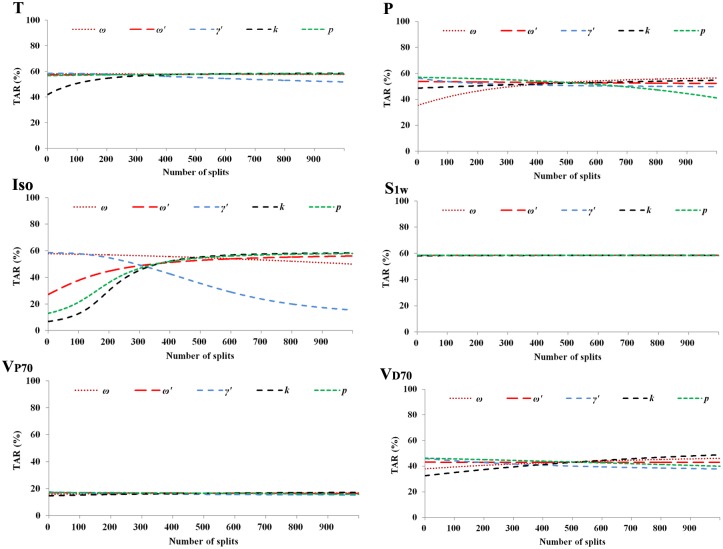
Sensitivity analysis of the parameters *ω*, *ω'*, *γ'*, *k* and *p* based on the outbreak in 2009. The numbers of splits of the five parameters (*ω* = 0.53, *ω'* = 0.83, *γ'* = 0.24, *k* = 0.5, and *p* = 0.42) in our study were 614, 972, 263, 500 and 415, respectively. TAR: total attack rate; Iso: isolation; T: therapeutics; P: prophylactics; V_P70_: 70% of individuals vaccinated prior to the outbreak; V_D70_: 70% individuals vaccinated each day during the outbreak; S1w: school closure for one week.

**Fig 6:**
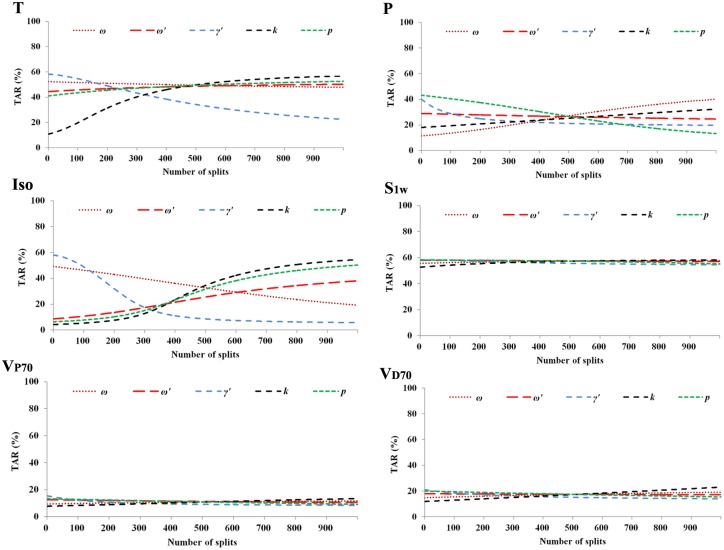
Sensitivity analysis of the parameters *ω*, *ω'*, *γ'*, *k* and *p* based on the outbreak in 2013. The numbers of splits of the five parameters (*ω* = 0.53, *ω'* = 0.83, *γ'* = 0.24, *k* = 0.5, and *p* = 0.42) in our study were 614, 972, 263, 500 and 415, respectively. TAR: total attack rate; Iso: isolation; T: therapeutics; P: prophylactics; V_P70_: 70% of individuals vaccinated prior to the outbreak; V_D70_: 70% individuals vaccinated each day during the outbreak; S1w: school closure for one week.

Figs 5 and 6 showed that the effectiveness of S1w, V_P70_ and V_D70_ were fairly stable to the change of parameters *ω*, *ω'*, *γ'*, *k* and *p*. Differently, T was sensitive to *γ'* and *k*. P was sensitive to *ω*, *γ'*, *k* and *p*. Iso was very sensitive to the change of all the five parameters.

## Discussion

Mathematical model has become a commonly used method to estimate the transmissibility of influenza and to assess the effectiveness of the countermeasures. Before the H1N1 pandemic occurred in 2009, Longini et al [14, 15] have employed a stochastic individual-based model to assess the effect of interventions like quarantine, antivirals and pre-vaccination for controlling the potential influenza pandemic. During the early stage of H1N1 pandemic in 2009, Yang et al [16] used a mathematical model to estimate the transmissibility of the virus. More other mathematical model research focusing on influenza pandemic can be found in literatures [5, 9, 13]. There are also a few researches focused on influenza outbreak in school [1, 21]. But to our knowledge, this is the first study which systematically assessed the effect of interventions of an influenza A (H1N1) outbreak in mainland China.

### Validity of the model

In our study, the SEIAR models were employed to fit the epidemic curves of two randomly selected outbreaks at school, the results of the Chi square test showed high good-of-fitness of our models with no intervention to the reported data, suggesting that that SEIAR models were suitable for this study and can be used to estimate the transmissibility of the outbreak at school and to assess the effectiveness of the countermeasures. The results of sensitivity analysis showed that the values of the parameters we set in our study might affect the effectiveness of T, P and Iso, the change of these parameters did almost not affect the effectiveness of the other three interventions, even though some of them were from previously publications.

### The proportion of asymptomatic

Patrozou and Mermel [22] reported that asymptomatic infection played an important role in the transmission of influenza, which could significantly reduce the effectiveness of countermeasures. Compared to those studies [14, 15] which obtained the proportion of asymptomatic from literatures, we used epidemiological serosurvey to estimate the parameter in our models. The results of a study showed that the proportion of asymptomatic could be as high as up to 70% [23]. Asymptomatic of influenza A (H1N1) also occurred in children infection [24]. In our study, we found that the proportion was 41.54% in Changsha city, China, after analyzing the results of 1500 blood sample. Therefore, we must determine the proportion of asymptomatic in an outbreak before making a control strategy.

### The effectiveness of interventions and their implications to practice

In our study, we selected two influenza A (H1N1) outbreak randomly from all collected outbreaks as typical events to evaluate the effectiveness of interventions by the mathematical model we built. Through our modeling, we found that the model with no intervention well fitted the data before interventions were taken. The combined intervention T+P+Iso+S1w+V_P70_+V_D70_ could be the best measure when *p* = 41.54%. However, with the exception of isolation, other interventions are, to some extent, difficult to implement in an outbreak. For example, problems associated with implementation of antivirals (therapeutic and prophylactic use) include high cost, risk of resistence and side-effect.

Oseltamivir is the only choice for prophylactic use, because influenza virus is highly resistent against adamantine. However, the cost of oseltamivir is high. The average cost of prophylactic is 150 RMB per day per person with the dose of 1 capsule for 10 days as a complete course by using the Oseltamivir manufactured in China. If the imported medicine is employed for the same prophylactic program, the cost would be 260 RMB per day per person. For prophylactic use, the coverage rate of the intervention must be high enough (such as the whole class, whole grade or whole school which an influenza case located) to ensure the effectiveness of the measure. Given the side-effects, resistance to prophylactic use of antivirals is higher than that therapeutic use of antivirals. For these reasons, Oseltamivir is rarely used in controlling an outbreak. The main problems of school closure are associated with operation of the school, care of children, and the risk of transmission in community when all the asymptomatic infection individuals come back to the community. There are two main issues with vaccination. First, it is difficult to increase the coverage of vaccination before an outbreak. Second, protective immunity from the vaccine is achieved only in about 10 days after the vaccination, and during this period vaccinated students remain susceptible upon exposure, therefore, we need to do a lot of risk communication to lessen the misunderstanding of parents of the cases.

Therefore, we need to overcome plenty of difficulties if we implement all the 6 counter-measures. From the results of our study, we can see that the TAR of P+Iso+S1w+V_P70_+V_D70_ is the same as T+P+Iso+S1w+V_P70_+V_D70_, and it resolves the problem of therapeutic of Oseltamivir, although it prolongs 4–5 days of DO. The combined intervention P+Iso+S1w+V_P70_ could solve the issue brought by vaccination during the outbreak, although the TAR is a little higher and the DO is 1 or 5 days longer than P+Iso+S1w+V_P70_+V_D70_. Similarly, the combined intervention Iso+S1w+V_P70_ could solve the issue brought by prophylactic during the outbreak, although the TAR is a little higher and the DO is several days longer than P+Iso+S1w+V_P70_. Interestingly, V_P70_ is included in these optimized strategies. Thus, we recommend strongly that the coverage of influenza vaccine should be higher than 70% of school-age children. Nowadays, the system of checking the immunization record has been built in China to focus on the children entering a kindergarten or primary school. We recommend that influenza vaccine should be enrolled in this checking system, and all the children should be vaccinated before their register except the contraindication.

In the outbreak in 2013, we can see that the result of Iso+S1w+V_D70_ could be good enough for controlling an outbreak, because it could reduce TAR to 3.83% (95%CI: 3.75%–3.91%) and DO to 39 days. The TAR of S1w+V_D70_ and Iso+ V_D70_ are 6.12% (95%CI: 6.03%–6.21%) and 8.32% (95%CI: 8.21%–8.43%), and the DO of them are 45 days and 50 days, respectively. We also found that the TAR would be high up to 23.20% (95%CI: 23.03%–23.37%) with all non-vaccination interventions. Therefore, vaccination with high coverage could be the key intervention to prevention and control an influenza A (H1N1) outbreak. If the coverage of the vaccine is not high enough before the outbreak begins, it is vital that vaccination during an outbreak could be an effective intervention. Similar results are observed in the outbreak in 2009 despite a difference in the most effective two-combined intervention (Iso+P). Such a difference may be due to different transmissibility, population and reporting time of the two outbreaks.

In our randomly selected outbreak example in 2013, the combined intervention Iso+S1w was implemented for 5 days firstly, then Iso was only employed for 3 days for final exams of the semester, then Iso+S1w was recalled because of the winter vacation. In this case, that the TAR could be controlled down to 3.20% (95%CI: 3.13%–3.27%) benefited from the winter vacation, because the TAR would be high up to 24.33% (95%CI: 24.16%–24.50%) if we only chose Iso+S1w according to our simulation. Normally, the duration of school closure could not be longer than one week during a small-scale outbreak in school. Similar simulation results are observed in the outbreak in 2009. Thus, it is not suitable to employed Iso+S1w in an outbreak.

### Limitations

There are several limitations in our study. First, the interaction of school and community as well as the differential protective effects were not considered in our model. Second, because of lack of relevant evidence, we assumed the vaccine efficacy against susceptibility (VE_*S*_), the vaccine efficacy against pathogenicity or symptomatic illness (VE_*P*_) and the vaccine efficacy against infectiousness (VE_*I*_) as 100%, which may deviate from the reality to some extent. Last, our findings are sensitive to the change of some parameters. Especially, the effectiveness of T, P, and Iso is sensitive to the change of two, three and all of the five parameters, respectively. Thus, the implications of our findings should be limited to the range of parameter approaching to the values of this study.

## Conclusions

In conclusion, an immunologic barrier should be built in children entering kindergarten and primary school for controlling an influenza A (H1N1) outbreak in which the asymptomatic infection could be high up to 41.54%. If the immunologic barrier could not reach 70% before an outbreak occurs, vaccination during the outbreak should be strongly recommended, which should be accompanied by isolation of all cases and closure of school for one week.

## Supporting information

S1 File Timing of the influenza A (H1N1) cases in an outbreak in 2009. (XLSX)

S2 File Timing of the influenza A (H1N1) cases in an outbreak in 2013. (XLSX)
